# Barriers to searching for grey literature in systematic reviews: A survey of authors and editors

**DOI:** 10.1017/rsm.2026.10087

**Published:** 2026-05-12

**Authors:** Jane O’Sullivan, Sarah Dawson, Julian Piers Thomas Higgins

**Affiliations:** 1 Anaesthesia and Critical Care, University Hospital Limerick, Ireland; 2 Department of Population Health Sciences, University of Bristol Population Health Sciences, UK; 3 Population Health Sciences, University of Bristol Medical School, UK; 4 Population Health Sciences, University of Bristol, UK

**Keywords:** grey literature, review methodology, search strategy

## Abstract

To explore the perceptions of, and barriers to, grey literature searching among medical researchers and journal editors. A cross-sectional survey of authors of systematic reviews and the editors of the journals in which the reviews were published. Systematic reviews indexed in MEDLINE, spanning a 4-week period in 2019. We excluded protocols. We asked whether the reviewers were performing a grey literature search. If they were, we asked about their approach to the grey literature search and relevant guidance. If they were not, we asked about their rationale for this. We elucidated understandings of grey literature from all reviewers. The survey to journal editors asked about their perceptions towards grey literature. A consecutive sample of 1,229 systematic reviews was included. A total of 155 authors responded, and a total of 46 journal editors responded. The majority (57%) of reviewers reported performing a grey literature search. However, there was no consensus on types of grey literature items or sources among the reviewers. The most frequent barrier to grey literature searches was concern about the quality/detail of the grey literature items. Editors expressed negative perceptions towards grey literature, rooted in suspicion of its quality. While many reviewers reported performing a grey literature search, there was a diverse understanding of the term grey literature. Concerns about the quality of grey literature exist among reviewers and editors alike. There is a marked discrepancy between the best-practice guidelines and the gatekeepers of journals.

## Highlights

### What is already known on this topic?

- Systematic reviewers are generally encouraged to perform a grey literature search.
- There is considerable confusion about how a grey literature search ought to be performed for a systematic review.

### What is new?

- Among 155 review authors and 46 journal editors surveyed, definitions and practices relating to grey literature varied substantially.
- Although 57% of review authors reported performing a grey literature search, many used sources unlikely to retrieve grey literature comprehensively.
- The most frequently reported barrier was concern about the quality or level of detail in grey literature items, and many journal editors expressed reluctance to accept non-peer-reviewed or unpublished material.

### Potential impact for RSM readers

- Systematic review authors exhibit inconsistent definitions and practices for grey literature, leading to variability in search strategies.
- Key barriers include concerns over quality, resource intensiveness, and misaligned expectations from journal editors regarding grey literature.
- There is a clear need for standardised, structured guidance to harmonise grey literature searching and improve its integration in systematic reviews.

## 1 Introduction

Grey literature, sometimes referred to as ‘fugitive literature’, has traditionally been defined as information produced outside conventional publishing and distribution channels, often lacking formal peer review. This definition was formalised in the Luxembourg Definition (1997) and was later expanded in New York in 2004.^1^ While this foundational definition remains influential, recent literature has expanded on its practical implications and relevance to evidence synthesis. For example, Adams et al.^2^ provide critical reflections on grey literature use in public health reviews, and the Campbell Collaboration has issued detailed guidance on identifying, searching for, and documenting grey literature in systematic reviews.^3^ An alternative definition of grey literature has been as ‘manifold document types produced on all levels of government, academics, business and industry in print and electronic formats that are protected by intellectual property rights, of sufficient quality to be collected and preserved by library holdings of institutional repositories, but not controlled by commercial publishers’.^4^ Further definitions exist.^5^

Whatever the definition, grey literature is recognised as a diverse body of material. Examples of grey literature include government reports, working papers, press releases, theses, and conference proceedings. It can represent anything from a dissertation to a government document to a blog.^6^ Given the diversity of formats and sources encompassed by grey literature, variation in how systematic reviews search for it and report it is expected. Thus, understanding grey literature can be extremely challenging.^2^

A review of existing guidance highlights the importance of comprehensive literature searching in systematic reviews.^7^ However, there is no consensus on how to perform a grey literature search consistently across reviews. The *Cochrane Handbook for Systematic Reviews of Interventions* recommends searching for sources of grey literature. It describes grey literature as reports published outside the traditional commercial publishers.^1^ Search strategies for comprehensive grey literature searches have been proposed.^8^^–^ ^11^ Existing guidance has attempted to address these issues, most notably in the Cochrane Handbook’s Technical Supplement to Chapter 4^1^^,^ ^12^ and the Campbell Collaboration’s recent recommendations for grey literature search strategies and reporting.^3^ However, there is no consensus on how to perform a grey literature search consistently across reviews, reflecting the heterogeneity of sources and approaches involved. Consensus may be more feasible at the level of specific types of grey literature rather than the category as a whole.

Despite the guidelines, a previous literature survey found that only a very small proportion of systematic reviews included unpublished literature.^13^ Another older study found that participants from grey literature studies contributed 11% of the study participants included in a sample of 135 meta-analyses.^14^ These low proportions may be explained by difficulties that researchers face when performing a grey literature search. While there is anecdotal evidence of such difficulties,^9^ there is no current evidence based on surveying reviewers about the collective difficulties that they face while performing a grey literature search. Moreover, a study more than 30 years ago determined that 30% of journal editors would not publish a meta-analysis with unpublished studies.^15^ However, there is a paucity of recent work to determine if this attitude still persists.

## 2 Objective

To determine what are the perceptions of, and barriers to, grey literature searching among medical researchers and journal editors.

## 3 Methods

We undertook a survey of authors of systematic reviews and the editors of the journals in which the reviews were published. We identified the systematic reviews in the context of an empirical study reported in detail elsewhere.^16^ In brief, we searched MEDLINE to capture all reports of systematic reviews indexed during a 4-week period (spanning October/November 2019) to capture a representative sample. Survey invitations were distributed in May 2020, and the survey remained open for 6 weeks. We included the reviews in our analysis if they satisfied the following criteria: (i) the report provided a clear statement of eligibility criteria for included studies; (ii) the review was ‘systematic’ in the sense of appearing to have searched comprehensively for studies meeting the eligibility criteria (either because this is stated directly or it can be inferred indirectly from the authors’ use of two or more databases); (iii) the report documented the search strategy, providing at a minimum the information sources for the review; and (iv) the report was published in English. The empirical study included 1,262 systematic reviews, of which 66 were Cochrane Reviews and 1196 were non-Cochrane reviews. For the present study, we excluded reviews reported only in protocols and a small batch of other records where the authors had only searched one database (Epistemonikos).

For each of the included systematic reviews, we recorded the corresponding author and journal of publication. We contacted each corresponding author via a personalised email, which cited the relevant systematic review. The email included a link to a survey via Survey Monkey, asking a series of questions about their understanding of searching for grey literature. If we received an automated error message, specifying that the email address of the corresponding author (as listed in the review) was no longer valid, we sought the most up-to-date email address elsewhere. The Cochrane and non-Cochrane authors received separate links to an identical survey. This enabled us to distinguish easily between the survey respondents as either Cochrane or non-Cochrane authors. The survey asked 14 questions which covered: the search process for the systematic review, understanding the meaning of ‘grey literature’, use of guidance on searching for grey literature, and what barriers might impact the search for grey literature (see Appendix A in the Supplementary Material).

We collated a list of all journals that had published the reviews in the sample and identified the (senior) journal editor. Where multiple journal editors were listed, we selected the content editor. We contacted each selected journal editor via email or via the online queries portal of the journal’s website, again providing a link to a survey via Survey Monkey. We sent a single survey to any editor who covered multiple different journals in our list. The survey asked five questions about the editor’s perceptions and acceptance of grey literature (see Appendix B in the Supplementary Material).

We produced simple descriptive summaries of the survey responses and present these mainly as bar charts. For the survey of authors, we present overall results as well as separate results for Cochrane and non-Cochrane reviews.

## 4 Results

**Figure 1:**
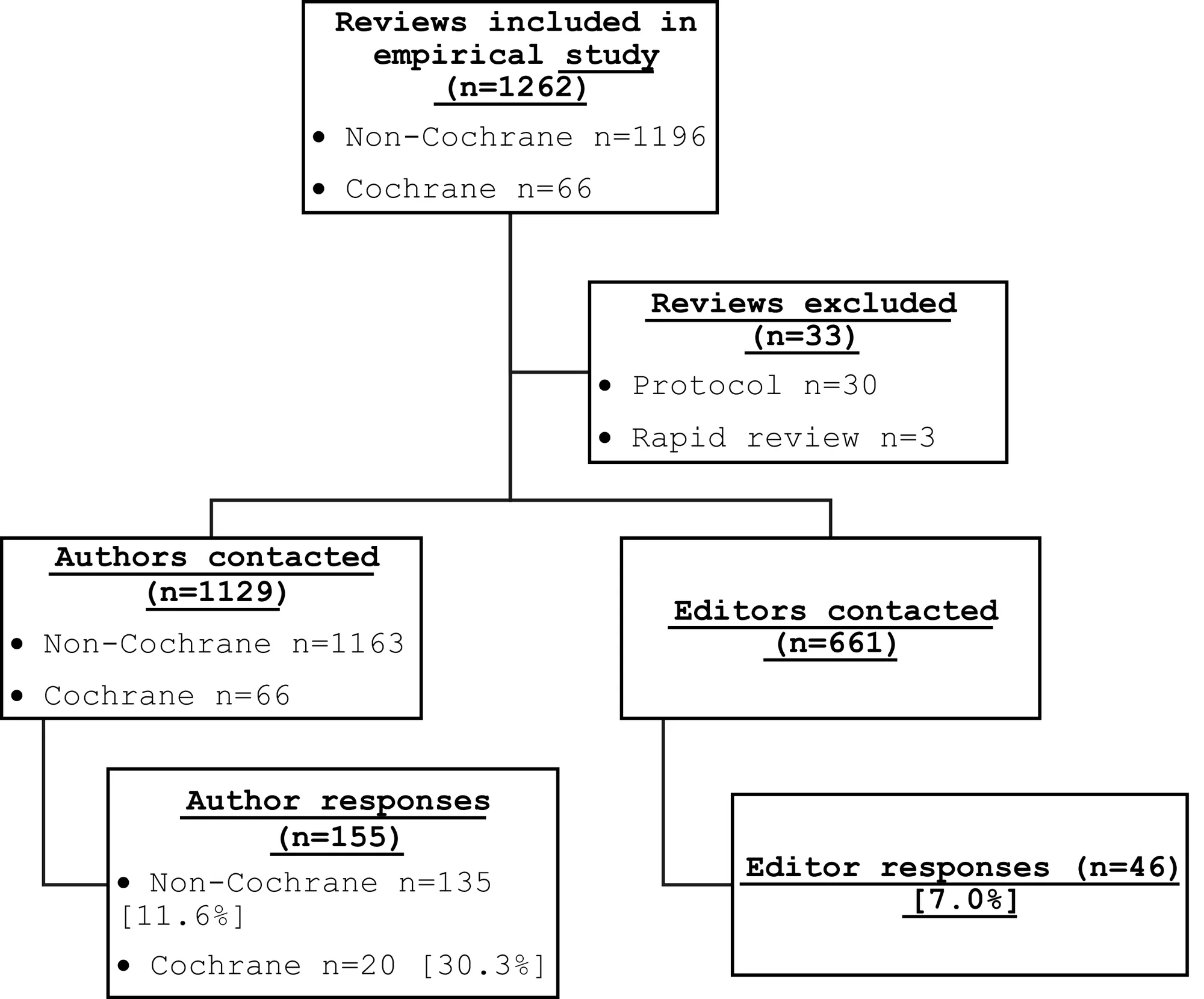
Flow diagram illustrating numbers of authors and editors contacted and completing the survey. Figure 1 Long description.

From the 1,262 reviews included in the empirical study, we excluded 33 protocols and rapid reviews (Figure 1), leaving 1,229 eligible reviews. We attempted to contact 1,229 authors and 661 editors (Figure 1). Of the eligible reviews, 155 authors and 46 editors completed the survey, corresponding to response rates of 12.6% and 7%, respectively. A breakdown of response rates by survey question and review type can be found in Appendix C in the Supplementary Material.

### 4.1 Review authors

**Figure 2:**
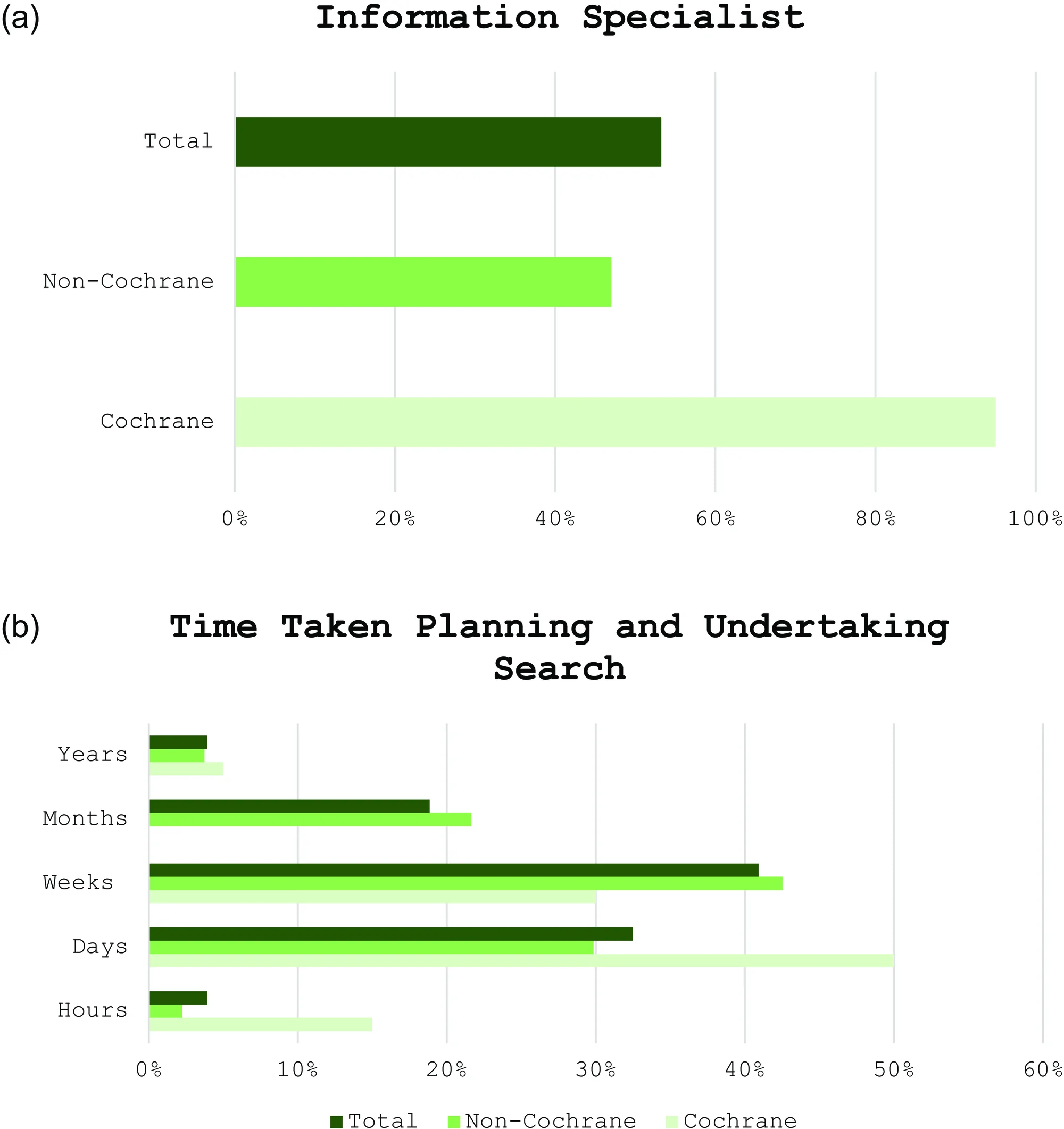
Planning and execution of the literature search: (a) the use of an information specialist for the planning and execution of the search; (b) the full-time equivalents of time taken to plan and undertake the search. Figure 2 Long description.

With respect to the search, 53% (82/155) of all reviewers who responded to our survey reported that they received assistance from an information specialist or librarian with 47% non-Cochrane and 95% Cochrane authors (Figure 2) (N.B. the proportion of Cochrane authors in our sample was small). The majority (43%) of non-Cochrane authors reported spending ‘weeks’ on the search strategy (rather than ‘hours’, ‘days’, ‘months’, or ‘years’), while the majority (50%) of Cochrane authors reported spending ‘days’ (Figure 2). The median number of non-Cochrane authors and Cochrane authors involved in the planning and execution of the search strategy was 3 and 2 authors, respectively.

**Figure 3:**
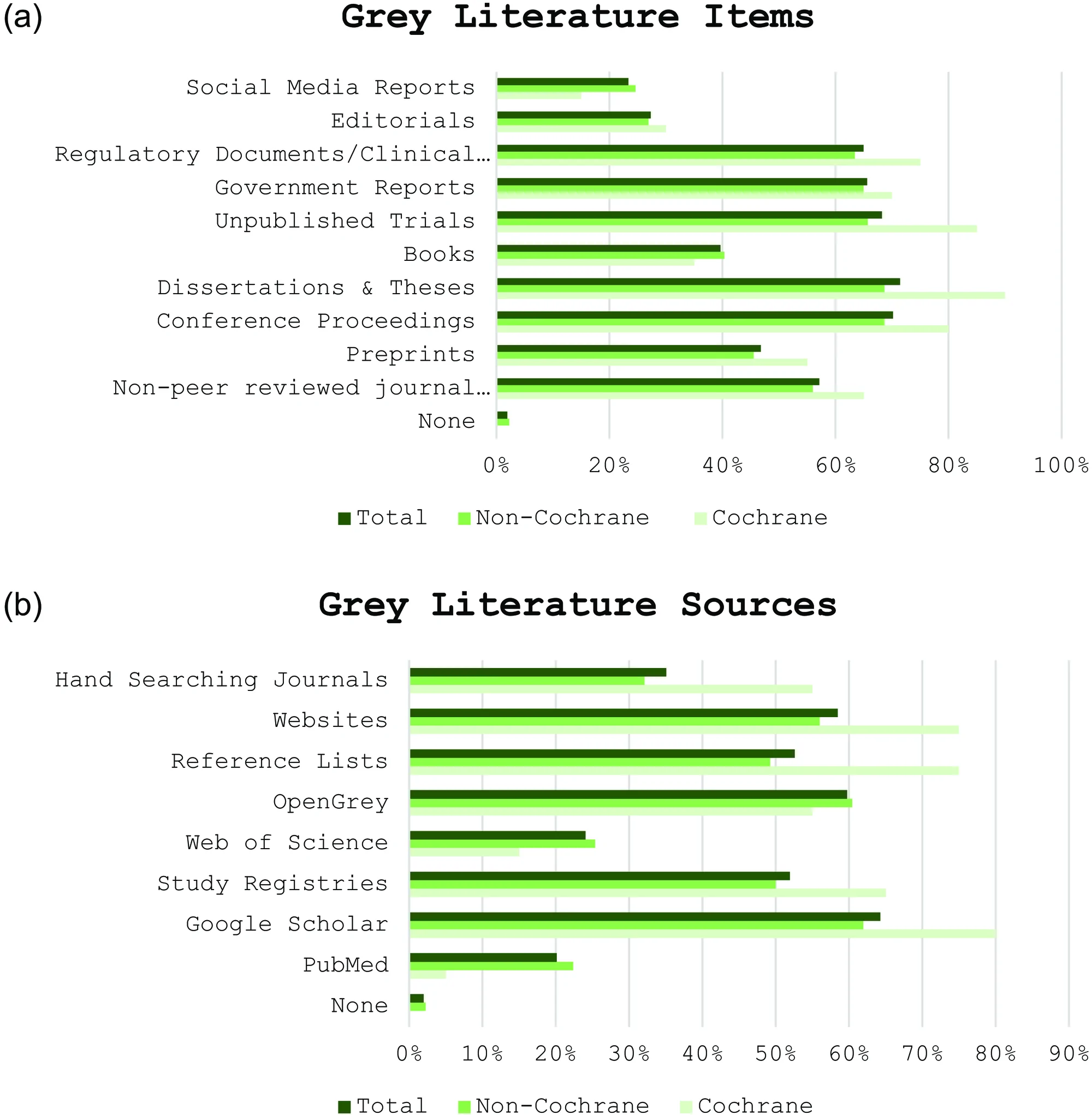
Understanding the meaning of grey literature by reviewers: (a) the items considered to be grey literature; (b) the information resources considered to be sources of grey literature. Figure 3 Long description.

The items most commonly considered by non-Cochrane authors to be grey literature were conference proceedings and dissertations (69% each). The majority of non-Cochrane authors also considered non-peer reviewed journal articles (56%), unpublished trials (66%), government reports (65%), and regulatory documents or clinical study reports (63%) to be grey literature items. Apart from books (35%), editorials (30%), and social media posts (15%), the majority of Cochrane authors thought all of the listed items were grey literature (Figure 3a).

The literature resource most commonly considered by non-Cochrane authors to be a source of a grey literature was Google Scholar (62%). The majority of non-Cochrane authors also considered study registries (50%) and Open Grey (60%) to be sources of grey literature. Apart from PubMed (5%) and Web of Science (15%), the majority of Cochrane authors considered all the listed resources to be sources of grey literature (Figure 3b).

**Figure 4:**
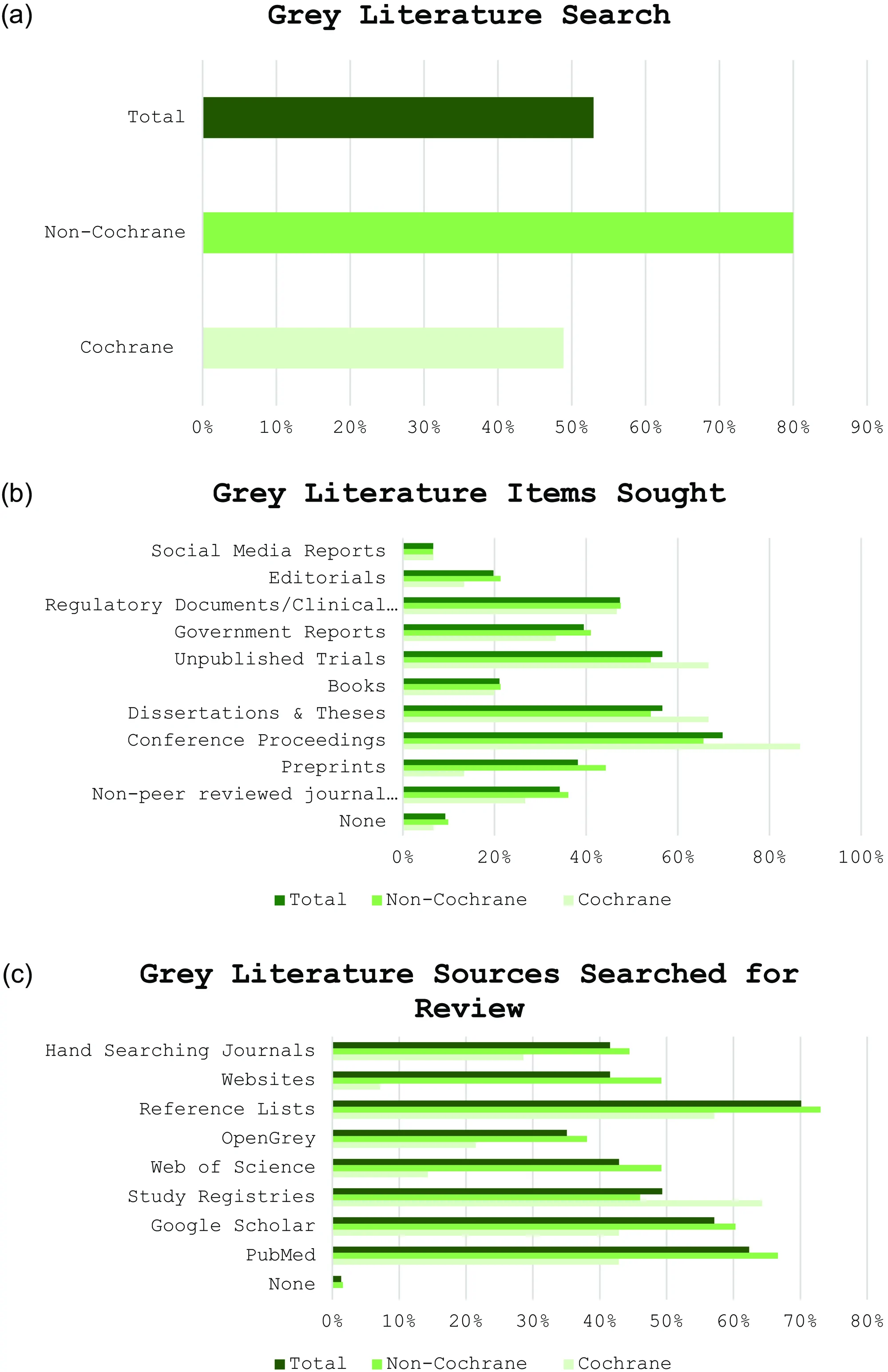
Conduct of a grey literature search: (a) performance of a grey literature search; (b) grey literature items sought within the reviews; (c) sources of grey literature searched by the reviewers. Figure 4 Long description.

A clear majority of Cochrane authors (80%) reported performing a grey literature search, compared with about half (49%) of non-Cochrane authors (Figure 4a). For those who reported performing a grey literature search, the majority of non-Cochrane authors sought the following grey literature items: conference proceedings (66%), dissertations/theses (54%), and unpublished trials (54%). Of the Cochrane authors who reported performing a grey literature search, the majority sought conference proceedings (87%), dissertations and theses (67%), and unpublished trials (67%) (Figure 4b).

For the non-Cochrane authors who said they performed a grey literature search, the majority of authors sought grey literature in reference lists (73%), PubMed (67%), and Google Scholar (60%). Of the Cochrane authors who said they performed a grey literature search, the majority sought grey literature in trial registries (64%) and reference lists (57%) (Figure 4c).

**Figure 5:**
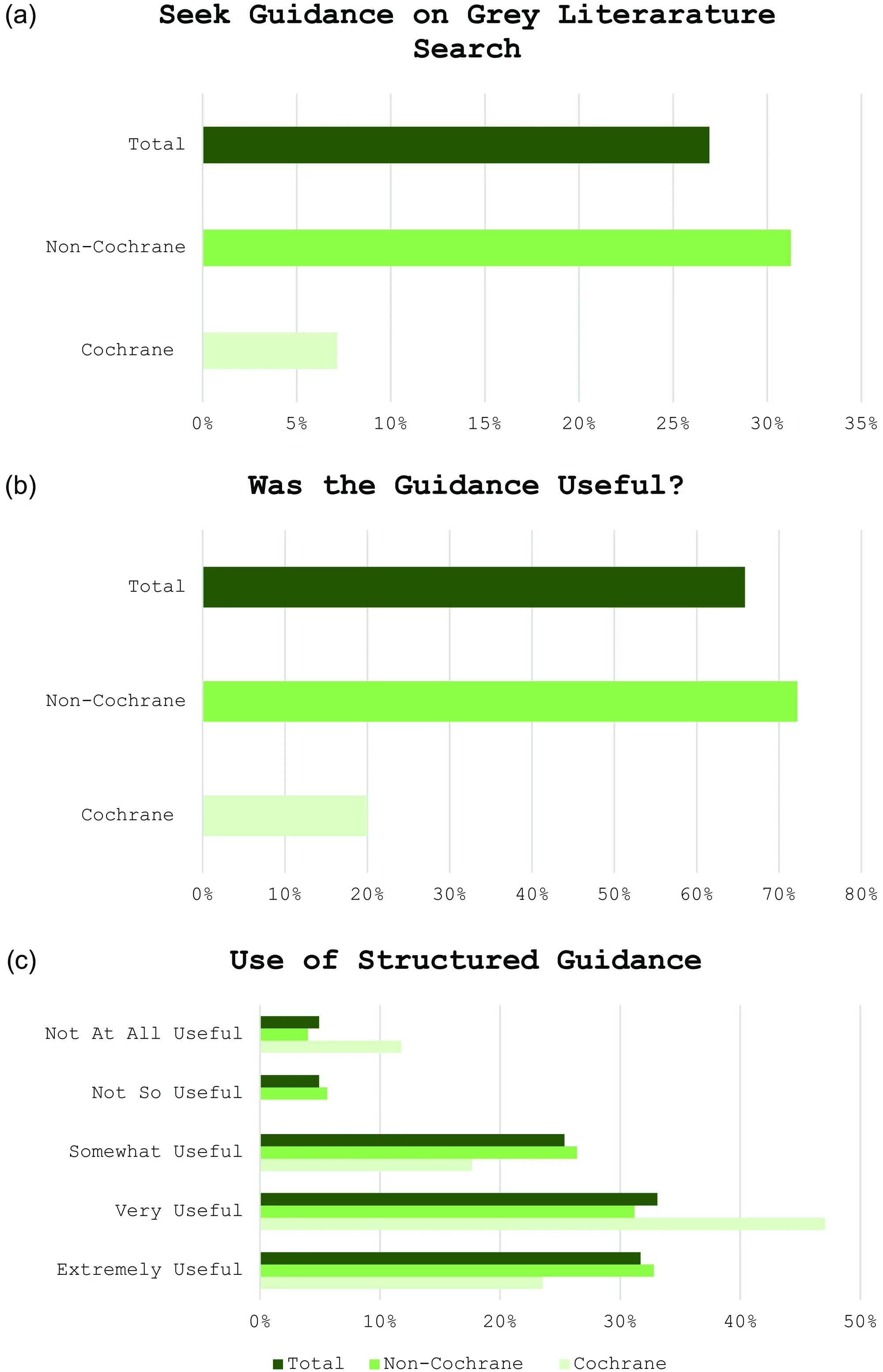
Use of guidance for grey literature search: percentage of authors who (a) sought guidance on their grey literature search; (b) found the written guidance on grey literature searching to be useful, among those who reported using any form of guidance; (c) would find structured guidance for performing a grey literature to be useful. Figure 5 Long description.

We asked the authors who reported performing a grey literature search about whether they used written guidance. A minority of non-Cochrane (31%) and Cochrane (7%) authors sought written guidance (Figure 5a). Of those who sought guidance, the majority of respondents sought guidance in the Cochrane Handbook (see Table 1). The majority (72%) of non-Cochrane authors found the guidance useful. Only a minority (20%) of Cochrane authors found the guidance useful (Figure *5*b). The majority of non-Cochrane (64%) and Cochrane (71%) authors reported that structured guidance would either be extremely useful, very useful, or somewhat useful (Figure 5c).

**Table 1:** Responses from authors to the question ‘What source of guidance was sought?’ Table 1 Long description.

	Number of authors (*n* = number of respondents)	
Source of guidance	Non-cochrane	Cochrane
Cochrane Handbook	6	1
Other books about systematic reviews (including the JBI Handbook)	3	0
PRISMA guidelines	2	0
Institutional instructions	1	0
Librarian	4	0
Academic personnel	4	0
Previous experience	3	0
Previous journal article	1	0
Other	4	1
N/A	5	2

**Figure 6:**
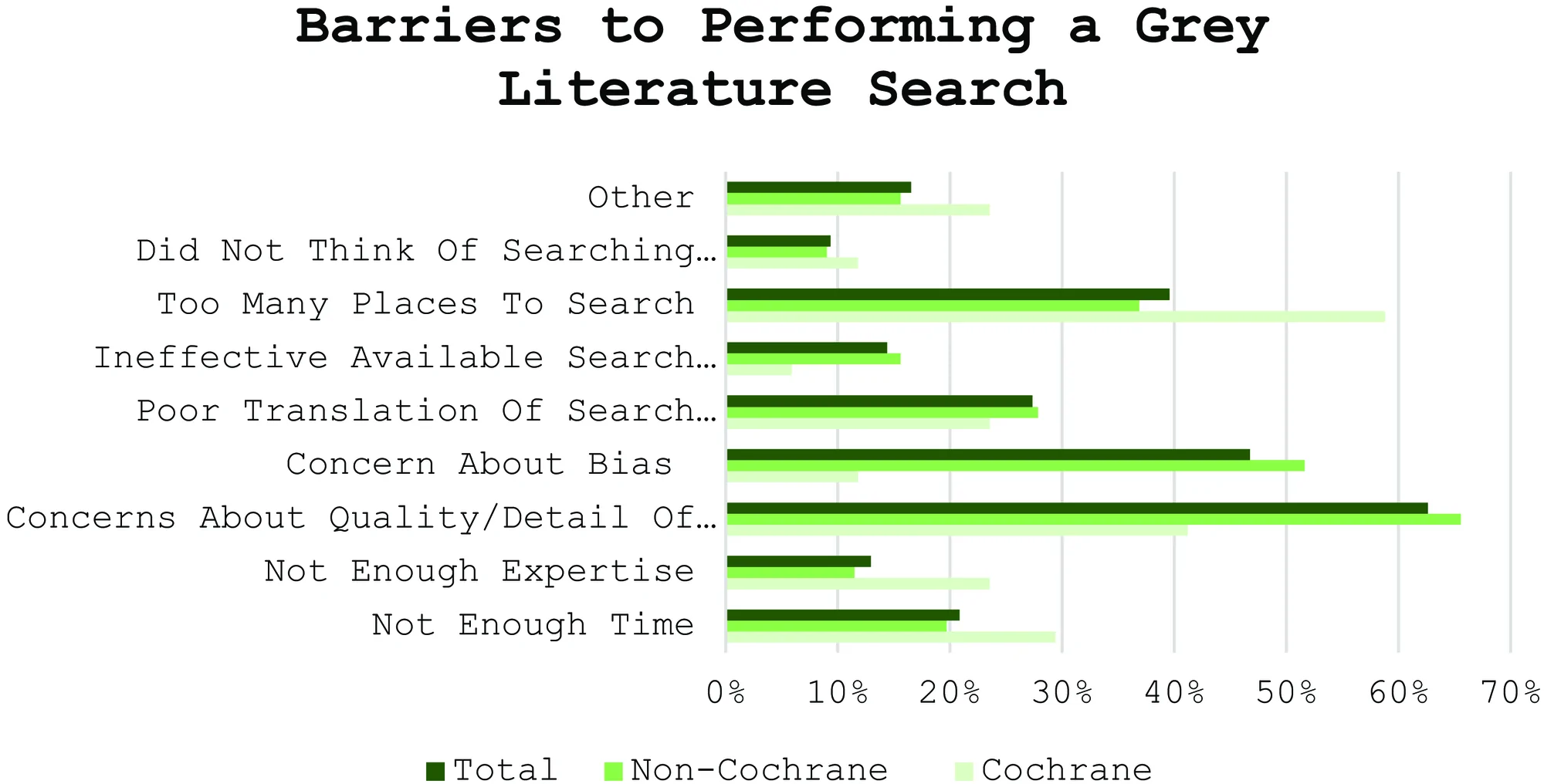
What were the barriers to performing a grey literature search. Figure 6 Long description.

The majority of non-Cochrane authors reported concerns regarding the quality/detail of reporting in grey literature (66%) and the potential for bias in studies reported in grey literature items (52%). The majority of Cochrane authors (58%) reported concerns regarding the number of possible sources of grey literature (Figure 6). Appendix D in the Supplementary Material outlines the freeform responses regarding grey literature that were submitted by the survey participants.

### 4.2 Journal editors

Of the small number of journal editors who responded to our survey, 83% reported that the search strategy is scrutinised as part of their peer-review process. In response to a follow-up question, 50% of editors reported that grey literature searches were being performed in the systematic reviews submitted to their journals. However, 65% of journal editors reported that a grey literature search was not expected for the systematic reviews submitted to their journals. Moreover, 50% of journal editors reported that they would not accept a systematic review that contained non-peer reviewed items. Nearly half (43%) of journal editors reported that they would not accept a systematic review that contained unpublished items.

## 5 Discussion

In our survey of a consecutive sample of 1,229 authors of systematic reviews, 135 non-Cochrane and 20 Cochrane authors responded. In a second survey to elicit attitudes of journal editors to grey literature, 46 journal editors responded.

We observed discrepancies and a lack of clarity about what constitute grey literature items and resources, among both non-Cochrane and Cochrane authors. Even an item we would clearly regard as ‘grey’, such as a dissertation/thesis, was not considered to be a grey literature item by all review authors. Similarly, many authors failed to describe OpenGrey as a grey literature resource. It should be noted that OpenGrey is no longer an actively maintained resource, which may impact its future utility. The discrepant responses suggest that there is a general lack of consensus on how to classify grey literature items and resources. This may be explained by the lack of authors using guidelines to perform the grey literature search. The majority of authors expressed a desire for clear structured guidance on how to perform a grey literature search.

When probed, the majority of authors reported that they performed a grey literature search. Among those authors who did not perform a grey literature search, the most commonly reported reason was the concern about the quality of and/or level of detail reported in a grey literature item. While concerns about the credibility of grey literature persist, peer review is not a universal proxy for quality. Grey literature undergoes varying levels of scrutiny, from rigorous academic evaluation—such as doctoral theses reviewed by examination committees—to internal institutional reports or policy documents that may involve only limited oversight. As such, the internal validity and methodological quality of grey literature should be appraised within the systematic review process itself, rather than being assumed based solely on publication type or venue.

In 2013, a large survey of 2,184 Cochrane authors was performed, focussing on their experience of searching for grey literature.^17^ Their response rate of 36.9% was similar to our response rate from Cochrane review authors. Of the review authors, 75.3% of respondents reported performing a grey literature search. This corresponds with our own report of Cochrane authors who performed a grey literature search (80%). Our study additionally included non-Cochrane authors, who were excluded from this earlier study, and in fact comprised the large majority of our respondents.

The results of our survey show that there is misunderstanding in the nature of grey literature and the conduct of a grey literature search. This confirms the observations made in previous studies on grey literature.^9^^–^ ^11^ Although the most commonly accepted definition of grey literature is as print or electronic information not controlled by commercial or academic publishers,^18^ there appears to be considerable variation in awareness of what constitutes grey literature among reviewers. This can possibly be explained by fluidity in the meaning of grey literature among information specialists.^18^

The information specialist appears to be an underused resource in the conduct of non-Cochrane reviews. One of the main roles of information specialists, especially in the context of healthcare research, is to support systematic reviews.^19^ Of the authors in our survey who reported performing a grey literature search, conference proceedings were the items most commonly sought. In a previous study, conference abstracts or unpublished data (trial registers, file drawer data, data from trialists) were the most common grey literature items included in the systematic reviews.^20^ Many review authors, including non-Cochrane and Cochrane authors, used PubMed as part of the grey literature search. While some non-journal documents, such as US government agency reports (e.g. Agency for Healthcare Research and Quality [AHRQ]), may be included in PubMed, it is unlikely that authors will encounter many grey literature items within this resource.

In the case of both non-Cochrane and Cochrane reviews, our respondents reported that a considerable amount of time was dedicated to the search strategy. In a previous study, 81 respondents who detailed their search strategy reported a median time of 471 minutes spent searching for studies for their systematic review.^21^ Moreover, a scoping review found that the median time for the search process to identify eligible studies for systematic reviews was between 7.85 and 22 hours. Additionally, the median time for the additional development of a search strategy for systematic reviews was between 1 and 13.5 hours.^22^ While our survey respondents reported spending much more time on their search strategy, this difference may be explained by the fact that their survey only asked about the amount of time spent searching and ours asked about both the planning and execution of the search strategy. Equally, the scoping review mentions reported outcomes in systematic reviews, whereas this study directly surveyed the authors.

There are several barriers to conducting searches for grey literature. The most, commonly cited barrier in this study was apprehension about the internal validity of the research identified. This corresponds with the previous findings by Saleh et al, which identified the lack of peer review and the quality of the individual studies as the greatest concern of review authors and journal editors.^21^ The rationale for excluding studies that are not peer reviewed is because peer-review is seen as a proxy for research quality.^23^ Our view is that the authors of a systematic review should be able to conduct an assessment of internal validity at least as effectively as a peer reviewer, such that restriction to peer-reviewed literature should not be necessary. However, we suspect that there are many systematic review teams without such skills. A relevant point is that some items of grey literature are insufficiently detailed to enable full assessment of the study quality. To date, we are unaware of any study that empirically examines the internal validity of studies reported in the grey literature versus non-grey literature.

Half of journal editors in our survey reported that review authors are performing a grey literature search as part of their systematic review. This is similar to the proportion of authors who report having performed a grey literature search. The majority of journal editors did not expect a grey literature to be performed as part of a systematic review. This conflicts with the current recommendation in the Cochrane Handbook, which advocates a grey literature search be performed where eligible and appropriate.^1^ In a 2006 survey, a total of 93 journal editors responded, and 70% of these felt that grey literature probably or definitely should be considered as part of a systematic review.^24^ However, a much older study in 1993 reported that only 47% of journal editors expected grey literature to be included in the systematic review.^15^ The discrepancy may be due to the fact that in 1993, Cochrane was in its infancy and the number of systematic reviews published before 1993 would have been very small in comparison with today.^25^ A lot of methodological initiatives, by Cochrane and other organisations, have been created to ensure that reviewers minimise reporting bias when conducting a search. It remains unclear whether there is an ongoing discrepancy between current guidance and the expectations of the journal editors in this present study.

Editors are the gatekeepers of their journals.^26^ Our findings suggest that resistance to the inclusion of non-peer reviewed and unpublished materials may have increased rather than diminished over time. Whereas a study conducted more than 30 years ago reported that approximately 30% of journal editors would not accept a meta-analysis including unpublished studies, our findings indicate that nearly half of editors surveyed would not accept a systematic review containing non-peer reviewed items, and 43% would not accept one containing unpublished studies. This highlights the persistence, and potential strengthening, of editorial barriers to comprehensive evidence synthesis.

A key strength of our study is its cross-sectional nature, with the survey being sent to authors of all systematic reviews published over a 4-week period in October/November 2019. While the overall response rate was low, a considerable number of authors still completed our survey. It highlights some of the misunderstanding of what is grey literature, among review authors. It further examines the apprehensions certain authors possess about including grey literature. The authors seem to express a desire for more structured guidance on the topic. Finally, it highlights the prejudices that editors have against grey literature.

There are limitations to this study. First, the response rate was low, as might be expected for a survey of this nature. It is unclear whether the authors received the emails but did not read them (no delivery notifications or read receipts were requested in the emails sent) or whether the emails were captured as spam. We suspect that authors who failed to respond to our survey were less likely to have performed a grey literature search. Thus, this current survey may not be representative of all authors in the included sample of systematic reviews. Second, while the Luxembourg and Prague definition are widely used to define grey literature,^4^^,^ ^18^ there is no universally accepted, working definition of grey literature or moreover a grey literature search.^27^ Hence, the survey respondents might not be expected to have the same shared understanding of grey literature. Finally, the survey was conducted in 2020 and, given the rapidly evolving nature of information retrieval methods, results may not reflect current practice.

This study suggests some future areas for research. There is a need for better understanding of the internal validity of studies reported in grey literature. There is also a need to learn more about the ability to assess internal validity based on grey literature items compared with non-grey literature such as journal articles. A structured tool or guidance tailored to each of the various forms of grey literature would also be of value to systematic review authors. In addition to developing new tools, increased awareness of existing resources—such as the Cochrane Handbook’s Technical Supplement—would likely enhance authors’ ability to identify and use grey literature effectively.

In conclusion, the search for (and inclusion of) grey literature is deemed highly desirable by the Cochrane Handbook. However, there appears to be a misunderstanding about the nature of grey literature, both in terms of what constitutes grey literature and where it can be found. While a slight majority of authors in our survey reported seeking grey literature for their review, they often sought it in resources unlikely to include grey literature items. There appears to be a need for clear, structured guidance on grey literature in the context of systematic reviews (what, where, and how to find it), which the respondents to our survey appear to endorse. Existing resources such as the Cochrane Handbook’s Technical Supplement to Chapter 4 provide detailed recommendations on grey literature searching and reporting, and wider awareness and uptake of such resources may help address this gap. We recommend that future systematic reviews explicitly state which types of grey literature were sought (e.g. theses, conference proceedings) and potentially adopt a checklist approach to improve transparency.

## Supporting information

10.1017/rsm.2026.10087.sm001 O’Sullivan et al. supplementary material O’Sullivan et al. supplementary material

## Data Availability

Data availability is not applicable to this article as no new data were created or analysed in this study.

## Supplementary material

To view supplementary material for this article, please visit http://doi.org/10.1017/rsm.2026.10087.

## Long descriptions

### Figure 1 Long description

The flowchart begins at the top with a central box: Reviews included in empirical study (n=1262), consisting of Non-Cochrane n=1196 and Cochrane n=66.

A vertical line descends to a side branch on the right: Reviews excluded (n=33), consisting of Protocol n=30 and Rapid review n=3.

Below the exclusion branch, the main path splits into two parallel columns.

Left Column (Authors):

• Authors contacted (n=1129), consisting of Non-Cochrane n=1163 and Cochrane n=66.

• A line leads down to Author responses (n=155), consisting of Non-Cochrane n=135 [11.6%] and Cochrane n=20 [30.3%].

Right Column (Editors):

• Editors contacted (n=661).

• A line leads down to Editor responses (n=46) [7.0%].

Navigate back to Figure 1.

### Figure 2 Long description

Panel a, titled Information Specialist, is a horizontal bar chart with a y-axis listing three categories and an x-axis showing percentages from 0 to 100 percent. The Cochrane group shows the highest involvement at approximately 95 percent. The Non-Cochrane group is at approximately 47 percent. The Total involvement is approximately 53 percent.

Panel b, titled Time Taken Planning and Undertaking Search, is a horizontal bar chart with a y-axis listing time units from top to bottom: Years, Months, Weeks, Days, and Hours. The x-axis shows percentages from 0 to 60 percent. Each time unit has three bars: dark green for Total, medium green for Non-Cochrane, and light green for Cochrane.

* Years: All groups are below 5 percent.

* Months: Non-Cochrane is highest at approximately 22 percent, followed by Total at 19 percent, and Cochrane at 0 percent.

* Weeks: Non-Cochrane is highest at approximately 43 percent, Total at 41 percent, and Cochrane at 30 percent.

* Days: Cochrane is highest at 50 percent, Total at 33 percent, and Non-Cochrane at 30 percent.

* Hours: Cochrane is highest at 15 percent, Total at 4 percent, and Non-Cochrane at 2 percent.

Navigate back to Figure 2.

### Figure 3 Long description

Panel a is titled Grey Literature Items. The y-axis lists eleven categories, and the x-axis measures percentage from 0 to 100 percent. A legend indicates three groups: Total (dark green), Non-Cochrane (medium green), and Cochrane (light green). From top to bottom, the items are:

* Social Media Reports: approximately 23 percent Total, 25 percent Non-Cochrane, 15 percent Cochrane.

* Editorials: approximately 27 percent Total, 27 percent Non-Cochrane, 30 percent Cochrane.

* Regulatory Documents/Clinical...: approximately 65 percent Total, 63 percent Non-Cochrane, 75 percent Cochrane.

* Government Reports: approximately 66 percent Total, 65 percent Non-Cochrane, 70 percent Cochrane.

* Unpublished Trials: approximately 68 percent Total, 65 percent Non-Cochrane, 85 percent Cochrane.

* Books: approximately 40 percent Total, 40 percent Non-Cochrane, 35 percent Cochrane.

* Dissertations & Theses: approximately 72 percent Total, 69 percent Non-Cochrane, 90 percent Cochrane.

* Conference Proceedings: approximately 70 percent Total, 65 percent Non-Cochrane, 80 percent Cochrane.

* Preprints: approximately 47 percent Total, 46 percent Non-Cochrane, 55 percent Cochrane.

* Non-peer reviewed journal...: approximately 57 percent Total, 55 percent Non-Cochrane, 65 percent Cochrane.

* None: less than 2 percent for all groups.

Panel b is titled Grey Literature Sources. The y-axis lists nine categories, and the x-axis measures percentage from 0 to 90 percent. The legend remains the same. From top to bottom, the sources are:

* Hand Searching Journals: approximately 35 percent Total, 32 percent Non-Cochrane, 55 percent Cochrane.

* Websites: approximately 58 percent Total, 56 percent Non-Cochrane, 75 percent Cochrane.

* Reference Lists: approximately 52 percent Total, 49 percent Non-Cochrane, 75 percent Cochrane.

* OpenGrey: approximately 60 percent Total, 61 percent Non-Cochrane, 55 percent Cochrane.

* Web of Science: approximately 24 percent Total, 25 percent Non-Cochrane, 15 percent Cochrane.

* Study Registries: approximately 52 percent Total, 50 percent Non-Cochrane, 65 percent Cochrane.

* Google Scholar: approximately 64 percent Total, 62 percent Non-Cochrane, 80 percent Cochrane.

* PubMed: approximately 20 percent Total, 22 percent Non-Cochrane, 5 percent Cochrane.

* None: approximately 2 percent for all groups.

Navigate back to Figure 3.

### Figure 4 Long description

A three-panel figure with horizontal bar charts.

Panel a, titled Grey Literature Search, shows the percentage of reviews that performed a search. The Total group is at 53 percent, Non-Cochrane is at 80 percent, and Cochrane is at 49 percent.

Panel b, titled Grey Literature Items Sought, compares ten categories across the three groups. The x-axis ranges from 0 to 100 percent.

- Conference Proceedings is the most sought item for Cochrane reviews at approximately 87 percent.

- Unpublished Trials are sought by 67 percent of Cochrane reviews compared to 54 percent of Non-Cochrane reviews.

- Dissertations and Theses are sought by 67 percent of Cochrane reviews and 55 percent of Non-Cochrane reviews.

- Preprints are sought by 45 percent of Non-Cochrane reviews but only 15 percent of Cochrane reviews.

- Other categories include Social Media Reports, Editorials, Regulatory Documents or Clinical reports, Government Reports, Books, and Non-peer reviewed journals.

Panel c, titled Grey Literature Sources Searched for Review, compares nine sources.

- Reference Lists are the most common source, used by over 70 percent of Non-Cochrane reviews and 57 percent of Cochrane reviews.

- PubMed is used by 67 percent of Non-Cochrane and 43 percent of Cochrane reviews.

- Google Scholar is used by 60 percent of Non-Cochrane and 43 percent of Cochrane reviews.

- Study Registries are used by 64 percent of Cochrane reviews but only 46 percent of Non-Cochrane reviews.

- Other sources include Hand Searching Journals, Websites, OpenGrey, and Web of Science.

Navigate back to Figure 4.

### Figure 5 Long description

The image consists of three panels arranged vertically.

Panel a, titled Seek Guidance on Grey Literature Search, shows the percentage of authors who sought guidance. The x-axis ranges from 0% to 35%. The Total bar is at approximately 27%. The Non-Cochrane bar is the highest at approximately 31%. The Cochrane bar is the lowest at approximately 7%.

Panel b, titled Was the Guidance Useful?, shows the percentage of authors who found written guidance useful. The x-axis ranges from 0% to 80%. The Total bar is at approximately 66%. The Non-Cochrane bar is the highest at approximately 72%. The Cochrane bar is the lowest at 20%.

Panel c, titled Use of Structured Guidance, is a grouped horizontal bar chart showing perceived usefulness levels. The x-axis ranges from 0% to 50%.

* Extremely Useful: Non-Cochrane is highest at 33%, Total at 32%, and Cochrane at 24%.

* Very Useful: Cochrane is highest at 47%, Total at 33%, and Non-Cochrane at 31%.

* Somewhat Useful: Non-Cochrane is highest at 26%, Total at 25%, and Cochrane at 18%.

* Not So Useful: Non-Cochrane and Total are around 5%, with Cochrane at 0%.

* Not At All Useful: Cochrane is highest at 12%, Total at 5%, and Non-Cochrane at 4%.

Navigate back to Figure 5.

### Table 1 Long description

The table is organized into three columns: Source of guidance, Non-cochrane, and Cochrane. The data rows are as follows:

* Cochrane Handbook: Non-cochrane 6, Cochrane 1.

* Other books about systematic reviews including the J B I Handbook: Non-cochrane 3, Cochrane 0.

* P R I S M A guidelines: Non-cochrane 2, Cochrane 0.

* Institutional instructions: Non-cochrane 1, Cochrane 0.

* Librarian: Non-cochrane 4, Cochrane 0.

* Academic personnel: Non-cochrane 4, Cochrane 0.

* Previous experience: Non-cochrane 3, Cochrane 0.

* Previous journal article: Non-cochrane 1, Cochrane 0.

* Other: Non-cochrane 4, Cochrane 1.

* N / A: Non-cochrane 5, Cochrane 2.

Navigate back to Table 1.

### Figure 6 Long description

The horizontal bar chart features an x-axis representing percentages from 0 percent to 70 percent in 10 percent increments. The y-axis lists nine barriers. Each barrier has three bars: dark green for Total, medium green for Non-Cochrane, and light green for Cochrane.

* Concerns About Quality/Detail Of...: This is the most significant barrier. Non-Cochrane is highest at approximately 65 percent, Total at 63 percent, and Cochrane at 41 percent.

* Concern About Bias: Non-Cochrane is at 52 percent, Total at 47 percent, and Cochrane at 12 percent.

* Too Many Places To Search: Cochrane is highest at 59 percent, Total at 40 percent, and Non-Cochrane at 37 percent.

* Poor Translation Of Search...: Non-Cochrane is at 28 percent, Total at 27 percent, and Cochrane at 23 percent.

* Not Enough Time: Cochrane is at 29 percent, Total at 21 percent, and Non-Cochrane at 20 percent.

* Not Enough Expertise: Cochrane is at 23 percent, Total at 13 percent, and Non-Cochrane at 11 percent.

* Other: Cochrane is at 23 percent, Total at 16 percent, and Non-Cochrane at 15 percent.

* Ineffective Available Search...: Non-Cochrane is at 15 percent, Total at 14 percent, and Cochrane at 6 percent.

* Did Not Think Of Searching...: Cochrane is at 12 percent, Total at 9 percent, and Non-Cochrane at 9 percent.

Navigate back to Figure 6.
